# Body mass index of 16-year olds in urban Maseru, Lesotho

**DOI:** 10.4102/phcfm.v6i1.618

**Published:** 2014-12-17

**Authors:** Violet L. van den Berg, Lisemelo Seheri, Jacques Raubenheimer

**Affiliations:** 1 Department of Nutrition and Dietetics, University of the Free State, South Africa; 2 Department of Biostatistics, University of the Free State, South Africa

## Abstract

**Background:**

Overweight and/or obesity amongst children and adolescents is a global epidemic with health consequences that track into adulthood. No data are currently available regarding overweight/obesity amongst adolescents in Lesotho.

**Aim and setting:**

To assess the prevalence of overweight and/or obesity and the associated risk factors amongst 16-year olds in urban Maseru, Lesotho.

**Method:**

A cross-sectional descriptive study was conducted on a systematic sample of 16-year olds in grade four (*N* = 221; 56.6% girls) from randomly-selected schools in urban Maseru. Diet histories and data on lifestyle, physical activity and knowledge, attitudes and/or perceptions and practices regarding nutrition were obtained during structured interviews and body mass index (BMI) was determined.

**Results:**

Amongst these 16-year olds, 27.2% girls and 8.3% boys were overweight and/or obese based on World Health Organization cut-offs for BMI; 39.8% were insufficiently active or inactive; 6.4% used alcohol regularly; and 11.7% used tobacco. Whilst 28.1% reported no television watching/electronic gaming/computer usage (combined screen time) outside school, 23.6% reported ≥ 4 hours of combined screen time outside school. Most (91.4%) consumed < 3 servings of vegetables/day; 86.4% consumed < 2 servings of fruits/day; and 95.5% consumed < 2 servings of dairy/day. The majority consumed maize porridge (56.1%), bread (63.8%) and margarine/oil/fat (82.3%) daily and added sugar to their food (74.2%). Fruits, vegetables, dairy, meat, pulses and traditional foods were only consumed weekly or less often. Most bought from tuck shops (18.6% daily; 54.3% weekly). Various gaps in knowledge, perceptions and practices were identified that may benefit from educational intervention.

**Conclusions:**

The current study identifies westernised dietary and lifestyle changes, along with overweight and/or obesity, amongst 16-year old adolescents in Lesotho.

## Abstrak

Indice de masse corporelle chez les adolescents de 16 ans dans l'agglomération de Maseru, au Lesotho.

**Contexte:**

L'excès de poids/obésité chez les enfants et adolescents est une épidémie mondiale qui a des conséquences sur la santé qui se poursuivent à l’âge adulte. A l'heure actuelle il n'existe aucune donnée sur l'excès de poids/obésité chez les adolescents du Lesotho.

**Objectif et lieu:**

Evaluer la prévalence d'excès de poids/obésité et les facteurs de risques qui lui sont associés chez les adolescents de 16 ans dans l'agglomération de Maseru, au Lesotho.

**Méthode:**

On a effectué une étude descriptive transversale sur un échantillon systématique d'enfants de 16 ans de grade quatre (*N* = 221; 56.6% de filles) d’écoles sélectionnées au hasard dans l'agglomération de Maseru. Au cours d'entretiens structurés on a noté les antécédents alimentaires et les données sur le mode de vie, l'activité physique et les connaissances, attitudes/perceptions et pratiques relatives à la nutrition ainsi que l'indice de masse corporelle (BMI).

**Résultats:**

Parmi ces adolescents de 16 ans, 27.2% des filles et 8.3% des garçons étaient en surpoids /obèses selon les seuils de l'OMS pour l'IMC; 39.8% n’étaient pas assez actifs/inactifs; 6.4% buvaient de l'alcool régulièrement; et 11.7% ont fait l'usage de tabac. Alors que 28.1% ne regardaient pas la télévision/ n'utilisaient pas les jeux électroniques/ l'ordinateur (temps passé devant un écran) en dehors de l’école, 23.6% passaient ≥ quatre heures devant un écran en dehors de l’école. La plupart (91.4%) consommaient moins de trois portions de légumes par jour; 86.4% consommaient moins de deux portions de fruits par jour; et 95.5% consommaient moins de deux portions de produits laitiers par jour. La majorité consommait de la bouillie de maïs (56.1%), du pain (63.8%) et de la margarine/de l'huile/des corps gras (82.3%) chaque jour et ajoutait du sucre à leurs aliments (74.2%). Ils mangeaient des fruits, des légumes, des produits laitiers, de la viande, des légumes secs et de la nourriture traditionnelle chaque semaine ou moins souvent. La plupart d'entre eux achetait à manger au kiosque (18.6% chaque jour; 54.3% chaque semaine). On a identifié des lacunes dans leurs connaissances, perceptions et pratiques qui pourraient être améliorées par l’éducation.

**Conclusions:**

Cette étude identifie les changements dus à l'influence occidentale dans le mode de vie et l'alimentation des adolescents de 16 ans du Lesotho, ainsi que le surpoids et l'obésité.

## Introduction

The World Health Organization (WHO) recognises the epidemic of childhood overweight/obesity as being one of the most serious global public health challenges of the 21st century.^1^ Obesity in childhood and adolescence has been identified as a key predictor for obesity in adulthood, as well as the early onset of insulin resistance, hypertension, dyslipidemia, and type 2 diabetes.^1–2,3^ In addition, morbidity and mortality in the adult population are increased in individuals who were overweight as adolescents, even if they lose the extra weight during adulthood.^2, 3^

In the United States, national representative data gathered for the period 2008–2009 indicated that 33.0% of boys and 30.4% of girls aged two to 19 are overweight and/or obese.^4^ In the 25 countries constituting the European Union, the prevalence of overweight/obesity is currently 16% – 22% amongst seven- to 17-year olds.^5^ The epidemic also affects developing countries,^1, 6^ with the recent prevalence of overweight/obesity amongst five- to 19-year olds at 41.8% in Mexico, 22.1% in Brazil, 22.0% in India and 19.3% in Argentina.^6^ In addition, studies of secular trends indicate that the prevalence of overweight and/or obesity is escalating at an alarming rate, both in developed and in developing countries.^6^

In 2004, the International Obesity Task Force estimated that less than 1% of children aged five to 17 years were overweight and/or obese in the sub-Saharan African region.^6^ However, the South African Health of the Nation Survey for 2001–2004 found that 13.3% of boys and 22.3% of girls aged six to 13 years, were overweight and/or obese;^7^ whilst the 1st South African National Youth Risk Behaviour Survey (SAYRBS) for 2002 reported that 7% of boys and 25% of girls aged 13 to 19 years were overweight/obese.^8^ When the SAYRBS was repeated in 2008, 11.2% of boys and 27.8% of girls adolescents (13 to 19 years old), were found to be overweight and/or obese.^9^ More recently, the South African National Health And Nutrition Examination Survey (SANHANES-1) found that 10.2% of boys and 22.3% of girls aged 10 to 14 years fell into this classification.^10^

The development of childhood overweight and/or obesity is attributed to a complex interplay between genetics and environmental factors.^11^ In developing countries, as a result of‘ aggressive advertising practices, relatively low cost of energy-dense foods and improved purchasing power’,^6^ children and adolescents are increasingly abandoning more prudent traditional diets ‘in favour of foods high in saturated fat and refined carbohydrates, sweetened carbonated beverages and diets low in polyunsaturated fatty acids and fiber’.^6,11^

Simultaneously, because of increased indoor leisure activities and entertainment, unsafe neighbourhoods and lack of open spaces in communities, these children and adolescents are also becoming increasingly more sedentary.^6^ Studies show that these specific patterns of nutrition and lifestyle transition are strongly associated with urbanisation and are highly conducive to the development of obesity, insulin resistance, cardiovascular diseases and type 2 diabetes.^6^, (^12^)

Moreover, in developing countries, intrauterine energy and nutrient deprivation are common amongst food-insecure mothers, also leading to metabolic adaptations in the foetuses which predispose them to future overweight and related metabolic disturbances, particularly when exposed during childhood to the above-mentioned transitional diet and lifestyle.^13^

The problem of overweight and obesity amongst children and adolescents in developing countries is further complicated by false traditional beliefs about and low knowledge of health and nutrition, as well as by specific socio-cultural perceptions regarding nutrition, gender roles and overweight and/or obesity amongst parents, caregivers and school children.^6^ Whilst various studies have focused on urbanisation, nutrient and lifestyle transition and overweight/obesity and their related health risks in the South African pediatric and adolescent population,^12,14,15^ few studies to date have investigated these problems in other sub-Saharan African countries.

The Kingdom of Lesotho is a small mountainous country of just over 30 300 km^2^, completely encircled by the Republic of South Africa. The climate is semi-arid with unpredictable weather patterns frequently characterised by droughts, heavy rainfall and hailstorms in summer, followed by very cold winters with frost and snow in the higher areas.^16^ The population of 1 936 181 (July 2013 estimate) consists of 99.7% Basotho.^17^ In 2010, 27% of the population lived in urban areas and it was estimated that the urban population will increase annually by 3.5% from 2010 to 2015.^16^ Around 35.0% of the population is aged below 15 years (2010 estimate).^16^

To date, however, no study has focused on overweight and/or obesity amongst children and adolescents in Lesotho. This study is therefore the first to report on overweight/obesity, the risk factors for weight gain, as well as knowledge, attitudes, perceptions and practices regarding overweight and/or obesity amongst adolescents (represented in this study by 16-year olds) in urban Lesotho. The study was restricted to one age group because the data were collected by a single researcher on a very limited budget. The age of 16 was chosen since national surveys often report overweight and obesity prevalence for adults in the population, namely, those over the age of 16 years. These data may serve to raise awareness of the emerging problem of overweight/obesity amongst the youth in Lesotho as a developing country where emphasis is placed on the prevention of undernutrition only; and to motivate future larger-scale surveys to assess the full extent of the problem amongst children and adolescents in the country. The results may also be used as baseline data to design intervention programmes to address the identified problems.

## Research methods and design

### Setting

The study was conducted amongst learners attending school in urban Maseru who were already 16 or had turned 16 in 2010. Maseru is the capital of Lesotho, located in the western low lands. It is by far the most populated city in the country (220 000 in 2010).^17^ In Lesotho, learners in urban areas start primary school at age 5 to 6 years. Primary education takes 7 years, followed by 3 years of junior secondary and 2 years of senior secondary education. In the Lesotho school system the majority of 16-year olds are in Form 4.

### Study population and sampling strategy

There are 20 schools in urban Maseru, of which 2 are private and 3 are single-gender. The private and the single-gender schools were included non-randomly in the sample. A further 5 schools were selected randomly from the remaining 15 mixed-gender public schools, so that a total of 10 schools was included in the study. There were 2510 students enrolled in Form 4 over these 10 schools for the 2010 school year. As coverage of a tenth of the population in the sample was considered both representative and practically achievable, a sample of 251 learners was selected to participate in the study. From each school, a number of learners proportionate to the size of the Form 4 class of that school, were chosen systematically from the admissions lists obtained from the various schools. Adolescents were eligible to be included in the study if they were 15 (turning 16 in 2010) or 16 years old and were in Form four in 2010. Adolescents were excluded from the study if they were aged 16 years, but not in Form 4 in 2010. A meeting was conducted with the selected learners at each school in order to inform them about the study. During these contact sessions, letters explaining the purpose of the study were handed out to all selected learners to read and to take home to their parents or legal guardians. Learners who wanted to participate in the study were required to return signed letters of consent from their parents or legal guardians and were also required to also sign letters giving their assent regarding inclusion. Learners retained the written information sheets.

### Data collection

Structured interviews were conducted in private with each participant in the participant's language of choice (English or Sesotho), in empty classrooms at the relevant schools. During the interview, weight and height were measured and questionnaires were used to collect data on socio-demography, dietary intakes, physical activity, screen watching habits, smoking habits and alcohol consumption, as well as knowledge, perceptions, attitudes and practices regarding nutrition. All data were collected by a single researcher who is a native of Maseru, Lesotho and is therefore familiar with the language and culture of the area; and who was trained in nutrition and the relevant research techniques at the University of the Free State in South Africa.

#### Anthropometry

During the interview, the researcher recorded the height and weight of each participant using standardised techniques^18^ and calibrated equipment.

#### Lifestyle factors

During the structured interviews, participants were asked: whether or not they consume alcohol; to indicate the types of alcoholic beverages they usually drink (red wine, white wine, beer, liquor or others which they could specify); to report how often they usually consume alcohol over a period of a week; and to indicate the volume of consumption of these different types of alcoholic beverages during the 7 days prior to the interview. In addition, participants were asked whether or not they had ever tried to smoke cigarettes or had ever used snuff; and to indicate on how many days in the 30 days prior to the interview they had smoked cigarettes or used snuff. As these may be sensitive questions for adolescents to answer since they are legally not permitted to purchase alcohol or tobacco products and are advised not to use these substances, the participants were reassured about the confidentiality with which the information would be treated. Participants were also asked to report the number of hours that they spend per day watching television, playing electronic games, using a computer or accessing the internet (which was noted as combined screen time).

Physical activity information was obtained with questions adapted from the SAYRBS^8,9^ which was in turn adapted specifically for South African adolescents from the International Physical Activity Questionnaire [IPAQ].^19^

#### Dietary intake and eating habits

In order to assess the overall balance of daily intake of foods from the basic food groups (starch; fruit; vegetable; milk and milk products; and meat and meat alternatives), as well as to quantify and evaluate daily intakes of energy and macronutrients, a 24-hour recall was administered during the structured interviews. Food photographs, packaging and local household utensils were used to assist participants in recalling portion sizes and food choices.

Food choices and dietary patterns were assessed by means of a non-quantified food frequency questionnaire (FFQ), which was administered during the same interview so as to determine how often particular foods within each food group were being consumed. The FFQ was adapted by the researcher to reflect foods from each of the food groups that are commonly used in Lesotho. Indigenous food choices such as wild fruits and traditional vegetable and traditional starch dishes were also included. An additional category included salty snacks, fast foods and beverages, as well as taking a packed lunch to school and buying from the school's tuck shop.

#### Knowledge, perceptions, attitudes and practices

Knowledge, perceptions, attitudes and practices regarding nutrition were recorded during the structured interviews by means of 49 questions adapted from the questionnaire used in the South African Food Consumption Survey Baseline for Food Fortification.^20^ The current questionnaire included: 12 questions to assess knowledge regarding selection of food items, ways in which foods are handled and methods in which foods are prepared (providing the options ‘true’, ‘false’ or ‘do not know’); 7 questions to assess attitude toward food and nutrition (providing the options ‘agree, ‘disagree or ‘do not know’); 9 questions to assess nutrition practices (providing the options ‘regularly, ‘occasionally or ‘never’); 10 questions to assess attitude toward obesity (providing the options ‘true’, ‘false’ or ‘do not know’); 6 questions to assess perceptions regarding the causes of obesity (providing the options ‘true’, ‘false’ or ‘do not know’); and 5 questions to assess knowledge regarding the relationship between obesity and health (providing the options ‘true’, ‘false’ or ‘do not know’).

### Data analysis

Reliability interviews were conducted one month after the initial interview on every 10th participant. If more than 20% of the answers to the questions differed between the 2 interviews, these questions were regarded as unreliable and excluded from the analysis. No questions were excluded.

Statistical analysis was performed by the Department of Biostatistics of the Faculty of Health Science of the University of the Free State and was generated by SAS® software 9.3 (SAS Institute, Cary, NC 2014). Categorical data were presented as frequencies and percentages; and continuous data as medians and percentiles.

Body mass index (BMI) was interpreted according to the WHO Z-score expanded tables which classify BMI-for-age as overweight if greater than + 1 standard deviation (SD) of the mean (equivalent to BMI 25 kg/m^2^ at 19 years) and as obese if greater than +2SD of the mean (equivalent to BMI 30 kg/m^2^ at 19 years).^21^ However, as these charts only became available in 2006, the CDC (US Centers for Disease Control and Prevention) data tables for BMI-for-age^22^ and the International Obesity Task Force (IOTF) age-specific cut-off-points^23^ for BMI at age 16 years were included so as to allow for comparison of the results with earlier studies in other countries. Pearson correlation analysis was used to evaluate associations between BMI and energy and macronutrient intakes (*p* ≤ 0.05 was considered significant).

Physical activity data were analysed according to the guidelines of the IPAQ.^19^ According to the IPAQ, ‘vigorous physical exercise comprises participation in activities that would make the participant sweat and breathe hard for at least 20 minutes’.^19^ For the purposes of the current study, participants were considered to have participated in sufficient vigorous physical activity if they reported having engaged in activities such as soccer, netball, rugby, basketball or running for at least 20 minutes on 3 or more days in the 7 days preceding the data collection. Moderate physical exercise comprises ‘participation in activities that would not make the participant sweat or breathe hard for at least 30 minutes’.^19^ In this study, participants were considered to have participated in sufficient moderate physical activity if they reported having engaged in activities such as walking, slow bicycling, skating, pushing a lawn mower, mopping, polishing or sweeping floors for at least 30 minutes on 5 or more days in the week preceding the data collection. Participants were considered to have been insufficiently active if they participated in some physical activity during the seven days preceding the data collection, but did not meet the above levels of activity. Participants were considered inactive if they did not report any participation in physical activity during the seven days preceding the data collection. For the purpose of this study, the frequencies were aggregated for participants who reported different combinations of both moderate and vigorous activity.

Usual daily food intakes were quantified from the record of usual daily intakes, using standard exchange lists compiled according to the recommendations of the American Dietetic Association^24^ and specifically adapted for South African and Lesotho food by quantifying portion sizes from the SA Medical Research Council's (MRC) Food Composition Tables^25^ and the SA MRC Food Quantity Tables.^26^ Intakes were thus expressed as servings from the different food groups and evaluated according to the recommendations of the United States Agricultural Department's Food Guide Pyramid 1992 (USAD-FGP),^27^ which quantifies the recommended daily number of portions to be consumed from each food group (6–11 from the starch group; 2–4 from the fruit group; 3–5 from the vegetable group; 2–3 from the milk and milk products group; and 2–3 from the meat and meat alternatives group). The exchange lists were also used to calculate the daily energy and macronutrient intakes, which were assessed compared to the estimated average requirements (EAR) for 16-year olds.^28^

The frequency with which a list of specific foods was included in the diet (recorded with the non-quantified FFQ) was expressed as the percentage of the participants that consume the individual food items on a daily, weekly, monthly or occasional basis, or never.

The answers to the questionnaire assessing knowledge, attitudes, perceptions and practices toward nutrition and obesity, were expressed as the percentage of the participants who chose the various answer options provided. The knowledge sections of the questionnaire were also scored according to the number of correct answers achieved.

### Ethical considerations

The study protocol was approved by the Ethics Committee of the Faculty of Health Sciences, University of the Free State (ETOVS NR 162/09). Permission to carry out the research was obtained from the Chief Inspector in the Ministry of Education in Lesotho and from the heads of the schools involved in the study. Participation in the study involved no risk to the participants. In cases where health risks were identified in a subject during the study, participants received free dietetic advice and counseling. All information in the study was kept strictly confidential. During the reporting of the results the focus was strictly on group trends so that individual information remains confidential within the study.

## Results

A total of 221 learners participated in the study, representing a response rate of 88.4% (7 participants did not bring consent from their parents or legal guardians; 15 were absent from school because of unpaid school fees at the time of the study; and eight withdrew from the study, citing busy schedules).

### Socio-demographic status

The final sample of 221 participants consisted of slightly more girls (*n* = 125, 56.6%) than boys (*n* = 96, 43.4%). All were Basotho; and the majority attended mixed-gender schools (*n* = 139, 62.9% mixed-gender schools; *n* = 82, 37.1% single-gender schools) and lived at home (*n* = 198, 89.6% at home; *n* = 23, 10.4% in school hostels) during the school term.

### Body weight status

Based on the WHO percentile charts for BMI, 8 (8.3%) of the boys and 34 (27.2%) of the girls were overweight and/or obese. More overweight and/or obese adolescents were identified by the WHO standards than with either the CDC percentile charts or the IOTF age-specific cut-off points (Table 1).

**TABLE 1a:** Body weight status of 16-year-old students from schools in urban Maseru.

Body weight (*N* = 221)	Males (*n* = 96)				Females (*n* = 125)			
	Overweight		Obese		Overweight		Obese	
	*n*	%	*n*	%	*n*	%	*n*	%
WHO†	5	5.2	3	3.1	28	22.4	6	4.8
CDC†	3	3.1	3	3.1	20	16.0	6	4.8
IOTF†	9	9.4	0	0.0	25	20.0	4	3.2

Note: BMI classified according to the following growth standards for 16-year olds.

†, World Health Organization (WHO) percentile charts.

‡, US Centers for Disease Control and Prevention (CDC) 2000 percentile charts.

§, International Obesity Task Force (IOTF) age-specific cut-off points.

**TABLE 1b:** Physical activity levels of 16-year-old students from schools in urban Maseru.

Self-reported physical activity levels (*N* = 221)	*n*	%
**Vigorously active:** Participants who participated in at least 20 minutes of activity that would make them sweat and breathe harder than normal on at least 3 days during the 7 days preceding the interview.	81	36.7
**Moderately active:** Participants who participated in at least 30 minutes of activity that would make them sweat or breathe hard on at least 5 days the 7 days preceding the interview.	52	23.5
**Insufficiently active:** Participants who participated in some physical activity during the 7 days preceding the interview, but did not reach the above levels of activity	58	26.2
**Inactive:** Participants who did not report any participation in physical activity during the 7 days preceding the interview	30	13.6

**TABLE 1c:** Lifestyle habits of 16-year-old students from schools in urban Maseru.

Approximate number of self-reported hours spent watching television or using a computer outside school hours (*N* = 221	TV		Computer		Combined screen time	
	*n*	%	*n*	%	*n*	%
None (has no TV and/or computer or does not use it)	67	30.3	188	85.0	62	28.1
1 hour/day	36	16.3	12	5.4	35	15.8
2 hours/day	50	22.6	10	4.5	43	19.5
3 hours/day	34	15.4	4	1.8	29	13.1
4 hours/day	24	10.9	3	1.4	24	10.9
≥ 5 hours/day	10	4.5	4	1.9	28	12.7

**TABLE 1d:** Lifestyle habits related to self-reported alcohol usages of 16-year-old students from schools in urban Maseru.

Self-reported alcohol usage (*N* = 221)	*n*	%
Non-drinker: Males/females who never consume alcohol	206	93.2
Occasional drinker: Males/females who consume < 1 unit/week	1	0.5
Low drinker: Males who consume 1–10 units/week; females who consume 1–7 units/week	8	3.6
Moderate drinker: Males who consume 11–21 units/week; females who consume 8–14 units/week	3	1.4
Heavy drinker: Males who consume > 21 units/week; females who consume >14 units/week	3	1.4

**TABLE 1e:** Lifestyle habits related to self-reported tobacco usage of 16-year-old students from schools in urban Maseru.

Self-reported tobacco usage (*N* = 221)	*n*	%
Participants who have never smoked cigarettes, not used snuff	190	86.0
Participants who only used snuff	5	2.3
Participants who smoked cigarettes before, but not during the past month	14	6.3
Participants who smoked cigarettes on ≥ 1 days in the past month	10	4.5
Participants who smoked cigarette ≥ 20 days in the past month	2	0.9

### Lifestyle practices

The lifestyle practices of the participants are combined in Table 1. For the 7 days preceding the data collection, just more than a third (*n* = 81, 36.7%) of the participants reported sufficient vigorous activity, whilst almost a quarter (*n* = 52, 23.5%) reported sufficient moderate activity. More than one in 3 participants reported insufficient activity (*n* = 58, 26.2%) or being inactive (*n* = 30, 13.6%) in the week prior to the study. No significant correlations were found between BMI category and level of physical activity.

The majority (*n* = 188, 85%) of the participants reported that they did not play electronic games, use computers or access the internet outside school hours, with 67 (30.3%) also reporting that they did not watch television outside school hours. Almost a third (*n* = 62, 28.1%) reported no combined screen time (watching television, playing electronic games, using computers or accessing the internet outside school hours), whilst 23.6% (*n* = 52) reported 4 or more hours of combined screen time per day outside school hours.

Although 206 (93.2%) participants reported no alcohol drinking behaviour, 6.4% (*n* = 14) reported drinking at least one to 10 units per week (boys) or one to 7 units per week (girls). The most consumed types of alcohol were beer or cider. One in 10 (*n* = 26, 11.7%) reported that they had smoked tobacco (*n* = 12, 5.4%) on at least one day in the month prior to the interview and a minority (*n* = 5, 2.3%) used snuff.

### Dietary intake

The mean (± SD) daily intakes for energy and macronutrient intakes, quantified from the records of usual daily food consumption, were slightly higher for boys than girls (Table 2). The mean energy intake of boys was only 53.9% of the recommended EAR, whilst that of the girls was 66.1% of the recommended EAR for this age group. The reported mean fat intake was at around 20% of total energy intake, whilst around two thirds of mean energy intake was contributed by carbohydrate.

**TABLE 2:** Energy and macronutrient intakes of 16-year-old students from schools in urban Maseru.

Daily intakes (mean ± SD)	Males (*n* = 96)	EAR males 14-18 yrs*	Females (*n* = 125)	EAR females 14-18 yrs*
Energy (kJ)	7178.9 ± 2349.1	13 298	6576.5 ± 1006.5	9946
Carbohydrate (g)(Expressed as% of total energy intake)	276.8 ± 53.1 (65.3%)	130	260.6 ± 49.9 (67.3%)	130
Protein (g)(Expressed as% of total energy intake)	69.1 ± 14.9 (16.3%)	46	62.0 ± 12.3 (16.0%)	52
Fat (g)(Expressed as% of total energy intake)	36.1 ± 17.0 (19%)	-	35.0 ± 13.9 (20.3%)	-

*, EAR (Estimated average requirements) of 14–18 year old males and females.^28^

In the whole group (*N* = 221), BMI had a significant and positive correlation with total daily energy intakes (*R* = 0.2; *p* = 0.01) and daily carbohydrate intakes (*R* = 0.3; *p* < 0.01); and showed a trend toward a positive correlation with daily protein intakes (*R* = 0.1; *p* = 0.09), but did not have a significant correlation with daily fat intakes (*R* = 0.1; *p* = 0.14).

Compared with the recommendations for healthy eating as depicted in the USDA FGP,^28^ the majority of the participants (Table 3) did not meet the minimum daily requirements for vegetables (*n* = 202, 91.4%), fruit (*n* = 192, 86.7%) and milk and milk products (*n* = 201, 91.0%), whilst 37.6% (*n* = 83) did not meet the minimum daily intakes of meat and meat alternatives. Most (*n* = 165, 74.7%) had higher than recommended daily intakes of starchy foods which agree with the high percentage of energy derived from carbohydrates.

**TABLE 3:** Daily intakes from the basic food groups of 16-year-old students (*N* = 221) from schools in urban Maseru compared to the USDA Food Guide Pyramid -recommendations?.

Intakes from the different food groups	Recommendations‡	*n*	%
**Starchy foods**			

Below recommendations	< 6 servings per day	2	0.9
Within recommendations	6–11 servings per day	54	24.4
High intakes	> 11 servings per day	165	74.7
**Vegetables**			

Below recommendations	< 3 servings per day	202	91.4
Within recommendations	3–5 servings per day	18	8.1
High intakes	> 5 servings per day	1	0.5
**Fruits**			

Below recommendations	< 2 servings per day	191	86.4
Within recommendations	2–4 servings per day	30	13.6
High intakes	> 4 servings per day	0	0.0
**Milk and milk products**			

Below recommendations	< 2 servings per day	211	95.5
Within recommendations	2–3 servings per day	10	4.5
High intakes	> 3 servings per day	0	0.0
**Meat and meat alternatives**			

Below recommendations	< 2 servings per day	83	37.6
Within recommendations	2–3 servings per day	45	20.4
High intakes	> 3 servings per day	93	42.1

USDA, U.S. Department of Agriculture;?, Evaluated according to the food groups and recommendations of the American Department of Agriculture's Food Guide Pyramid.^27^

These trends were confirmed by the FFQ (Table 4), in which the majority of participants indicated that they consumed maize porridge (*n* = 124, 56.1%), bread (*n* = 141, 63.8%) and margarine/butter/oil (*n* = 182, 82.3%); and that they added sugar (*n* = 164, 74.2%) and salt (*n* = 191, 86.4%), respectively, to food and/or drinks on a daily basis. Fresh or frozen vegetables and fruits, milk, cheese and yoghurt were mostly consumed on a weekly basis, or less often. Most participants reported that they never consumed wild vegetables (*n* = 138, 62.4%) or wild fruits (*n* = 185, 83.7%). Similarly, traditional foods such as *lesheleshele* [soft porridge made from sorghum meal], *motoho* [fermented soft porridge made from sorghum meal], *lepu* [stewed pumpkin leaves] and *mafi* [fermented milk], as well as traditional dishes (*nyekoe*, *potele*, *lipolokoe*, *seqhaqhabola* and *tsóeu-koto*), were consumed infrequently. Whereas meat and meat alternatives such as chicken, red meat, fish, eggs and pulses were only consumed weekly or less often, most participants reported that they consume high-fat processed meats such as bacon, polony, viennas, ham and Russians daily (*n* = 38, 17.1%) or weekly (*n* = 108, 48.9%). One in 4 (*n* = 57, 25.8%) reported that they consume sweets/chocolates daily; one in 5 (*n* = 45, 20.4%) that they consume packet chips (crisps) daily; and more than a third (*n* = 79, 35.7%) that they drink cordials/cordials/fizzy drinks daily. More than half (*n* = 113, 51.1%) reported that they never take a packed lunch to school and only about 6% (*n* = 13) that they take lunch daily, whilst most reported that they would rather buy from the schools’ tuck shops on a daily basis (*n* = 41, 18.6%) or weekly (*n* = 120, 54.3%). On the FFQ, almost a 10th (*n* = 18, 8.1%) of the participants reported using alcohol on a weekly basis, compared to the 6.4% (*n* = 14) that admitted to weekly intakes of alcohol on the lifestyle questionnaire. Most participants (*n* = 200, 90.5%) never used dietary supplements.

**TABLE 4:** Frequency of food consumption of 16-year-old students from schools in urban Maseru (*N* = 221).

Food and food groups	Daily		Weekly		Monthly		Occasionally		Never	
	*n*	%	*n*	%	*n*	%	*n*	%	*n*	%
**Breads, cereal, rice and pasta**										

Maize meal (stiff porridge / pap)	124	56.1	93	42.1	1	0.5	1	0.5	3	1.4
*Lesheleshele* (soft sorghum porridge)	11	5.0	53	24.0	41	18.6	6	2.7	110	49.8
*Motoho* (fermented sorghum meal porridge)	3	1.4	21	9.5	73	33.0	23	10.4	101	45.7
Bread Brown/White	141	63.8	76	34.4	2	0.9	0	0.0	2	0.9
Cereal	23	10.4	45	20.4	37	16.7	1	0.5	65	29.4
Rice/mealie rice	2	0.9	177	80.1	21	9.5	0	0.0	21	9.5
Samp	2	0.9	106	48.0	66	29.9	4	1.8	43	19.5
*Poone* (mealies)	1	0.5	8	3.6	9	4.1	160	85.5	34	15.4
Pasta	1	0.5	84	38.0	52	23.5	2	0.9	82	37.1
Popcorn	4	1.8	80	36.2	59	26.7	1	0.5	77	34.8
**Vegetables**										

Fresh/frozen	66	29.9	130	58.8	9	4.1	16	7.2	0	0.0
Wild vegetables	0	0.0	15	6.8	10	4.5	29	13.1	138	62.4
*Lepu* (pumpkin leaves)	0	0.0	0	0.0	0	0.0	136	61.5	85	38.5
Fruit										

Fresh/frozen	56	25.3	118	53.4	35	15.8	12	5.4	0	0.0
Wild fruits	1	0.5	4	1.8	6	2.7	25	11.3	185	83.7
Fruit juice	19	8.6	104	47.1	95	43.0	3	1.4	0	0.0
**Milk and milk products**										

Fresh milk	35	19.0	111	50.2	42	15.8	0	0.0	33	14.9
*Mafi* (fermented milk)	0	0.0	75	33.9	62	28.0	4	1.8	80	36.2
Cheese	6	2.7	56	25.3	47	21.3	0	0.0	109	49.3
Yoghurt	6	2.7	69	31.2	74	33.5	2	0.9	70	31.7
**Meat and meat alternatives**										

Chicken	2	0.9	173	78.3	46	20.8	0	0.0	1	0.5
Red meat	2	0.9	144	65.2	60	27.1	1	0.5	14	6.3
Bacon/Polony/Vienna/Ham/Russian	38	17.1	108	48.9	32	14.5	1	0.5	14	6.3
Fish	0	0.0	95	43.0	71	32.1	1	0.5	54	24.4
Eggs	12	5.4	140	63.3	35	15.8	0	0.0	34	15.4
Peas/Beans/Lentils/Soy beans	1	0.5	139	62.9	54	24.4	0	0.0	27	12.2
*Likahare* (Tripe / Offal)	0	0.0	51	23.1	88	39.8	11	5.0	71	32.1
Peanuts/Nuts	6	2.7	72	32.6	67	30.3	2	0.9	74	33.5
**Fats, oils and sugar**										

Peanut butter	11	5.0	87	39.4	41	18.6	3	1.4	79	35.7
Salad dressing/Mayonnaise	8	3.6	126	57.0	47	21.3	2	0.9	38	17.2
Coffee creamer	18	8.1	45	20.4	24	10.9	8	3.6	126	57.0
Butter/Margarine/Oil	182	82.3	28	12.7	5	2.3	2	0.9	4	1.8
Sweets/Chocolates	57	25.8	102	46.2	23	10.4	0	0.0	39	17.6
Add sugar to food/drinks	164	74.2	40	18.1	4	1.8	5	2.3	8	3.6
Jam	8	3.6	60	27.1	44	19.9	7	3.2	102	46.2
Cordials	79	35.7	88	39.8	30	13.6	0	0.0	24	10.9
Fizzy drinks (soda)	12	5.4	93	42.1	71	32.1	79	35.7	26	11.7
Ice cream	0	0.0	36	16.3	31	14.0	82	37.1	72	35.6
**Others**										

Add salt to food	191	86.4	14	6.3	2	0.9	2	0.9	12	5.4
Dry packet chips (crisps)	45	20.4	134	60.6	21	9.5	1	0.5	20	9.0
Take a lunchbox to school	13	5.9	91	41.1	4	1.8	0	0.0	113	51.1
Tuck shop	41	18.6	120	54.3	22	10.0	1	0.5	37	16.7
Traditional dishes	0	0.0	9	4.1	31	14.0	14	6.3	167	75.6
Beer/Cider/Wine/Liqueur	0	0.0	18	8.1	17	7.7	15	6.8	171	77.4
Dietary supplements	9	4.1	5	2.3	2	0.9	5	2.3	200	90.5

## Knowledge, perceptions, attitudes and practices regarding nutrition

Although most participants had high scores on the part of the questionnaire related to nutrition knowledge (Table 5, Questions 1 to 12; mean score 75% correct answers), certain topics for nutrition education were identified. Only half (*n* = 122, 55.2%) knew that soya mince was a healthy alternative to meat (Table 5, Question 8); only half (*n* = 109; 49.3%) knew that sugar is not a source of vitamins and minerals (Table 5, Question 6); just over a third (*n* = 82, 37.1%) knew that high alcohol consumption can cause weight gain (Table 5, Question 10); and only 30.8% thought that one should eat a lot of sugar in order to have energy (Table 5, Question 5).

**TABLE 5a:** Responses of 16-year-old students from schools in urban Maseru to questions related to knowledge regarding nutrition.

Knowledge of participants regarding nutrition (*N* = 221)	Options	*n*	%
1. Most nutrients are lost during the cooking.	True?	192	86.9
	False	20	9.0
	Do not know	9	4.1
2. Nutrient losses from fruits and vegetables can occur as a result of long storage.	True?	142	64.3
	False	49	22.2
	Do not know	30	13.6
3. Healthy foods help protect against illness.	True?	216	97.4
	False	5	2.3
	Do not know	0	0.0
4. Eating lots of different kinds of foods is healthier than eating a few kinds.	True?	170	76.9
	False	37	16.7
	Do not know	14	6.3
5. You should eat a lot of sugar to have energy.	True	68	30.8
\	False?	137	62.0
	Do not know	16	7.2
6. Sugar contains vitamins and minerals.	True	66	29.9
	False?	109	49.3
	Do not know	39	20.8
7. Dry beans, peas, lentils are a healthy choice to eat in place of meat.	True?	180	81.4
	False	26	11.8
	Do not know	15	6.8
8. Soya mince is as healthy as meat.	True?	122	55.2
	False	57	25.8
	Do not know	42	19.0
9. It is not healthy for pregnant women to drink alcohol.	True?	204	92.3
	False	16	7.2
	Do not know	1	0.5
10. Drinking a lot of beer and wine can make you put on weight.	True?	82	37.1
	False	111	50.2
	Do not know	28	12.7
11. Eat at least three meals a day.	True?	213	96.4
\	False	5	2.3
	Do not know	3	1.4
12. Eating breakfast every morning is healthy	True?	210	95.0
	False	7	3.2
	Do not know	4	1.8
**Mean number (%) of correct answers for questions 1 to 12 (*****N*** **= 221): 9.0/12 (75.0%)**			


†, Correct answer.

**TABLE 5b:** Responses of 16-year-old students from schools in urban Maseru to questions related to attitudes regarding nutrition.

Attitudes of participants regarding nutrition (*N* = 221)	Options	*n*	%
13. I only buy food that is good for my body.	Agree	124	56.1
	Disagree	85	38.5
	Do not know	12	5.4
14. I eat what I like, and do not care if it is healthy or not.	Agree	95	43.0
	Disagree	124	56.1
	Do not know	2	0.9
15. I have a right to buy foods that are healthy for my body.	Agree	216	97.7
	Disagree	5	2.3
	Do not know	0	0.0
16. I believe that the food I eat now will affect my health in future	Agree	137	62.0
	Disagree	57	25.8
	Do not know	27	12.2
17. I do not need to care about eating healthy to keep my weight constant.	Agree	28	12.7
	Disagree	187	84.6
	Do not know	6	2.7
18. I just do not have the time to think about food and nutrition.	Agree	67	30.3
	Disagree	141	63.8
	Do not know	13	5.9
19. I do not have to care about food unless the doctor or nurse talks about it.	Agree	36	16.3
	Disagree	181	81.9
	Do not know	4	1.8

**TABLE 5c:** Responses of 16-year-old students from schools in urban Maseru to questions related to practices regarding nutrition.

Practices of participants regarding nutrition (*N* = 221)	Options	*n*	%
20. Do you eat five vegetables and fruits a day?	Regularly	25	11.3
	Occasionally	128	57.9
	Never	68	30.8
21. Do you eat dry beans, split peas, lentils, soya twice or more/ week?	Regularly	72	32.6
	Occasionally	109	49.3
	Never	40	18.1
22. Do you drink water in between meals?	Regularly	156	70.6
	Occasionally	44	19.9
	Never	21	9.5
23. Do you drink cold drinks / fizzy drinks every day?	Regularly	41	18.6
	Occasionally	130	58.8
	Never	50	22.6
24. Do you eat cake, pastries or biscuits in between meals every day?	Regularly	17	7.7
	Occasionally	101	45.7
	Never	103	46.6
25. Do you add salt to your food at table?	Regularly	76	34.5
	Occasionally	81	36.8
	Never	64	28.6
26. Do you like to add sugar to vegetables in the cooking process?	Regularly	25	11.4
	Occasionally	54	24.5
	Never	142	64.1
27. Do you like to add butter/margarine/oil to cooked vegetables?	Regularly	12	5.5
	Occasionally	43	19.5
	Never	166	75.0
28. Do you often buy take-away foods?	Regularly	15	6.8
	Occasionally	88	40.0
	Never	118	53.2

The participants mostly agreed with statements depicting positive attitudes toward good nutrition (Table 5, Questions 13 to 19). The participants’ answers to questions regarding nutrition practices (Table 5, Questions 20 to 28) supported the results obtained from the records of usual daily intakes and the FFQ in portraying low intakes of vegetables and fruit (Question 20) and pulses (Question 21) and regular intakes of cold drinks/fizzy drinks (Question 23). Adding sugar and butter/margarine/oil to cooked vegetables did not seem to be a regular practice in this population.

## Attitudes, perceptions and knowledge regarding obesity

The participants had mixed attitudes toward obesity, as well as toward efforts to lose weight or to be thin (Table 6, Questions 29 to 39). Almost 70% (*n* = 152) felt that ‘fat people cannot work hard’ (Table 6, Question 32) and 40% (*n* = 88) that ‘fat people are unhappy’ (Table 6, Question 34). Regarding the acceptability of obesity, only 25 (11.4%) agreed with the statements that ‘fat people have more friends’ (Table 6, Question 30); more than half (*n* = 123, 55.5%) felt that ‘children do not like their mothers to be fat’ (Table 6, Question 31); and about a 5th (*n* = 42, 18.9%) felt that ‘men prefer fat women’ (Table 6, Question 33). Regarding weight loss, just under half (*n* = 98, 44.5%) felt that ‘it is difficult to lose weight’ (Table 6, Question 38) and about a third (*n* = 74; 33.6%) that ‘if one loses weight, one looks unattractive with loose skin’ (Table 6, Question 39). Only 18.6% (*n* = 35) of participants felt that ‘people who eat healthy foods are thin’ (Table 6, Question 35). Regarding physical activity, 92.3% (*n* = 204) agreed that ‘if one exercises daily (Table 6, Question 36), one feels healthy’ and 188 (85%) agreed that they ‘enjoy exercise’ (Table 6, Question 37).

**TABLE 6a:** Responses of 16-year-old students from schools in urban Maseru to questions related to attitudes regarding obesity.

Attitude of participants towards obesity (*N* = 220*)	Options	*n*	%
29. Fat people have more friends.	True	25	11.4
	False	118	53.6
	Do not know	77	35.0
30. Children do not like their mothers to be fat.	True	122	55.5
	False	54	24.4
	Do not know	44	20.0
31. Fat people cannot work hard	True	152	69.1
	False	57	25.9
	Do not know	11	5.0
32. Men prefer fat women.	True	41	18.6
	False	117	53.2
	Do not know	62	28.2
33. Fat people feel unhappy.	True	88	40.0
	False	64	29.1
	Do not know	68	30.9
34. People who eat healthy food are thin.	True	41	18.6
	False	155	70.5
	Do not know	24	10.9
35. If one exercises daily, one feels healthy.	True	203	92.3
	False	9	4.1
	Do not know	9	3.6
36. I enjoy bodily exercise.	True	187	85.0
	False	29	13.2
	Do not know	4	1.8
37. It is difficult to lose weight.	True	98	44.5
	False	98	44.5
	Do not know	24	10.9
38. If one loses weight, one looks unattractive with loose skin.	True	74	33.6
	False	107	48.6
	Do not know	39	17.7

**TABLE 6b:** Responses of 16-year-old students from schools in urban Maseru to questions related to perceptions regarding obesity.

Perceptions of participants regarding the causes of obesity (*N* = 220*)	Options	*n*	%
39. If one eats late in the evening, one is likely to get fatter.	True	60	27.3
	False	116	52.7
	Do not know	44	20.0
40. Fat on meat does not make one fatter.	True	64	29.1
	False	126	57.3
	Do not know	30	13.6
41. Lots of sugar in tea makes one fatter.	True	71	32.3
	False	113	51.4
	Do not know	36	16.4
42. Lots of oil and fat in food make one fatter.	True	173	78.6
	False	36	16.4
	Do not know	11	5.0
43. When one drinks a lot of water, one eats less.	True	107	48.6
	False	81	36.8
	Do not know	32	14.5
44. When one eats a lot of fat in food, one feels comfortable.	True	58	26.4
	False	129	58.6
	Do not know	33	15.0

**TABLE 6c:** Responses of 16-year-old students from schools in urban Maseru to questions related to knowledge regarding obesity.

Knowledge of participants regarding the relationship between obesity and health (*N* = 220?)	Options	*n*	%
45. Thin people get more sugar diabetes than fat people.	True	21	9.5
	False‡	151	68.6
	Do not know	48	21.8
46. More fat people than thin people suffer from high blood pressure.	True‡	177	80.5
	False	21	9.5
	Do not know	22	10.0
47. Thin people get tired easier than fat people.	True	7	3.2
	False‡	205	93.2
	Do not know	8	3.6
48. Fat people with sugar diabetes become healthier when they lose weight.	True‡	110	50.0
	False	47	21.4
	Do not know	63	28.6
49. Thin women do not get pregnant easily.	True	16	7.3
	False‡	156	70.9
	Do not know	48	21.8
**Mean number (%) of correct answers for questions 45 to 49 (*****N*** **= 220): 3.6/6 (59.6%)**			


†, One participant was unavailable to complete this part of the questionnaire during a structured interview.

‡, Correct answer.

Asked about some of the causes of overweight/obesity (Table 6, Questions 39 to 44), about a third (*n* = 71, 32.3%) thought that weight gain (‘getting fat’) may be caused by using ‘lots’ of sugar in tea (Table 6, Questions 41), or eating late in the evening (Table 6, Question 39) (*n* = 71, 32.3%; and *n* = 60, 27.3%, respectively). About half (*n* = 107, 48.6%) thought that drinking a lot of water could help one to eat less (Table 6, Question 43). Although most (*n* = 174, 78.6%) thought that ‘lots’ of fat/oil in food could cause weight gain (Table 6, Question 42) and most (*n* = 130, 58.6%) thought that eating a ‘lot’ of fat in food does not make one feel comfortable (Table 6, Question 44), a third (*n* = 64; 29.1%) did not think that eating fat on meat could cause weight gain and 30 (13.6%) did not know (Table 6, Question 40).

The last part of the questionnaire tested the participants’ knowledge and perceptions on the relationship between obesity and health (Questions 45 to 49). Not all participants related obesity to diabetes (*n* = 152, 68.6%) (Table 6, Question 45), hypertension (*n* = 178, 80.5%) (Table 6, Question 46) and infertility (*n* = 65, 29.4%) (Table 6, Question 49), whilst only half agreed that weight loss would be beneficial to people with diabetes (Table 6, Question 48). The mean knowledge score on this part of the questionnaire, calculated from the number of correct responses to questions 45 to 49, was 59.6%.

## Discussion

This study identifies overweight and/or obesity as an emerging health problem amongst Basotho adolescents in urban Lesotho, with 6% to 9% of 16-year-old boys and 21% to 27% of 16-year-old girls being overweight and/or obese, depending on the growth standards used. The study identified several known risk factors for adolescent overweight/obesity amongst these 16-year-old Basotho, including insufficient or no physical activity in almost 40%; more than 4 hours per day of combined screen time in almost a quarter (23.6%); regular alcohol consumption in 6.4%; and tobacco use in 14% of participants; as well as evidence of nutritional transition toward more unhealthy westernised eating habits. Some misperceptions regarding nutrition and obesity were also clear in a substantial proportion of the participants. This is significant as only a decade ago overweight/obesity was considered to be low in sub-Saharan countries compared with other developing countries.^6^ This concurs with a recent report describing increasing trends of overweight/obesity amongst children < 5 years in African countries, which may eventually track into adolescence.^29^ The majority of sub-Saharan countries, including Lesotho, however, have limited available representative data on overweight/obesity prevalence because public health and nutrition-related efforts have been focused mostly on undernutrition and food safety.^30^

In the current study, overweight and/or obesity was classified according to the WHO growth standards, the CDC growth standards and the IOTF age-specific cut-off points. The WHO growth standards,^21^ released in 2006, were developed based on longitudinal data of children from six different cohorts representing different cultures and ethnic settings around the world. The WHO aimed to develop a single international growth standard that represents the best description of physiological growth in all children, as well as to establish the breastfed infant as the normative for growth and development.^21^ The CDC cut-off points were developed based on five nationally-representative surveys of American children conducted between 1963 and 1994.^22^ The IOTF cut-off points were based on children living in six countries (i.e., United States, Brazil, Great Britain, Hong Kong, Netherlands and Singapore) and were extrapolated to the widely-accepted definitions for adult overweight and obesity of BMI ≥ 25 and BMI ≥ 30, respectively.^23^ Studies (none yet from Africa) comparing results using the three different standards report varying results.^31,32,33^ In the current study, the WHO standards in comparison with the CDC standards identified more of the 16-year-old boys and girls as overweight, but similar numbers as obese. By contrast, the IOTF cut-off points identified fewer obese boys and girls, but more overweight boys than the other two standards (Table 1). These findings highlight the importance of considering the definitions used to classify BMI when studying and comparing prevalence data for overweight and/or obesity between studies and countries.

The prevalence of overweight and/or obesity found in the current study amongst 16-year-old Basotho in urban Maseru is slightly lower than that reported for adolescents in surrounding South Africa. Using the IOTF cut-offs, 9.4% of boys and 23.2% of girls in the current study were identified as overweight/obese (Table 1). According to the SAYRBS2008 (which compared BMI to the IOTF cut-offs), 12.1% of black males and 36.2% of black females between 13 and 19 years old were overweight and/or obese.^9^ The prevalence of overweight and obesity amongst the Basotho adolescents was also lower than amongst American adolescents, particularly amongst boys. The USYRBS20114 (which compared BMI to the CDC growth standards) found that 30.5% of American black males and 38.2% of American black females in Grades 9 to 12, were overweight/obese, compared with 6.2% of the Basotho males and 20.8% of the Basotho females (when using CDC cut-offs and combining the overweight and obese categories; Table 1) in the current study. These results, therefore, point to emerging overweight/obesity amongst Basotho adolescents, represented in this study by 16-year olds in urban Maseru; a problem which to date has not been identified or targeted specifically in Lesotho.^30^

Lack of sufficient physical activity is one of the major risk factors for overweight and/or obesity in children and adolescents.^6,8,9^ Apart from assisting in weight control, adequate physical activity also reduces the risk for non-communicable diseases independently of weight.^34^ It has also been suggested that physically-active adolescents are more likely to adopt other healthy behaviour such as avoiding tobacco, alcohol and drug use, as well as demonstrating higher academic performance at school.^31^ Surveys in the United States and South Africa^9^ reported ethnic differences with regard to activity level. The current study found that combined insufficient and no physical activity (39.8%) was almost as common amongst these Basotho adolescents as amongst their South African counterparts (41.7% reported in the SAYRBS2008).^9^ However, only 36.7% of the Basotho participants participated in significantly vigorous activity in the week preceding the data collection, compared with43.0% of black South African adolescents.^9^ Comparison with the findings of the USYRBS2011,^4^ as well as with many other studies, is difficult because different standards were used to classify physical activity levels. However, sedentary behaviour seems to affect a substantial proportion of Basotho adolescents.

Extended screen time per day is considered to be a risk for childhood and adolescent overweight and/or obesity as it constitutes a form of sedentary behaviour.^4,8,9^ Compared with South African adolescents (29.3% nationally and 30% black South Africans),^9^ fewer Basotho adolescents in this study (23.6%), reported spending 4 hours or more per day of combined screen time (watching television, playing electronic games, using a computer or the internet) outside school hours, whilst almost a third reported no screen time outside school hours. The Basotho adolescents watched considerably less television than American adolescents. According to the USYRBS 2011,^4^ 32.4% of adolescents nationally and 54.6% of black adolescents watch more than 3 hours of television per day, compared with 15.4% of the Basotho adolescents who reported watching 4 hours or more of television per day. Similarly in the United States, 31.1% of adolescents nationally and 38.1% of black adolescents used a computer outside school or played video games for more than 3 hours per day,^4^ compared with only 3.3% of the Basotho adolescents who reported 4 or more hours of such activities outside school. A possible limitation of the current study (as well as in the USYRBS^4^ and SAYRBS^8,9^) is that social interaction on cellphones was not included as a form of sedentary behaviour, since adolescents may spend hours engaged in these activities.

Evidence from cross-sectional and prospective studies indicate that 3 or more units of alcohol per day (≥ 21 units per week) may contribute to overweight and obesity.^35^ Although only 1.4% of the Basotho adolescents reported drinking 14 to 21 units of alcohol per week (Table 1), excessive alcohol use independent of weight is also recognised as a major risk factor for non-communicable diseases.^34^ Furthermore, studies indicate that alcohol exposure in adolescence predicts heavy alcohol use later in life.^36^ Self-reported alcohol use amongst the Basotho adolescents in the current study was substantially lower than in the United States and surrounding South Africa. Of the Basotho adolescents, only 6.8% reported using any alcohol and 2.4% reported drinking more than a unit per week, compared to the United States where 38.7% of adolescents nationally (and 30.5% of black adolescents) reported consuming any alcohol in the month prior to the USYRBS2011,^4^ as well as South Africa where nationally 34.9% adolescents and 31.8% of black adolescents, reported consuming any alcohol in the month prior to the SAYRBS2008.^9^ However, the USYRBS^4^ and SAYRBS^8,9^ made use of anonymous self-reported questionnaires, whereas in the current study structured interviews were conducted with the participants, which may have resulted in under-reporting of their actual alcohol (and tobacco) use.

Tobacco use is the single largest cause of preventable death through non-communicable diseases in the world today.^34^ Smokers on average weigh less than non-smokers, because nicotine increases energy expenditure and can reduce appetite (making smoking attractive to adolescent girls).^37^ By contrast, however, heavy smokers tend to weigh more than light smokers or non-smokers, especially in persons of lower socio-economic status.^37^ Smoking causes hormonal imbalances which contribute to insulin resistance and obesity-related non-communicable diseases; in addition, smokers also tend to be more inactive and have a poorer diet, than non-smokers, both of which are conducive to weight gain.^37^ The majority of smokers start using tobacco before the age of 19 years.^9^ In the current study, 11.7% of the Basotho participants reported that they had ever tried smoking cigarettes in their lifetime (5.9% on at least more than one day in the month prior to the interview) and 2.3% reported using snuff. In South Africa, the SAYRBS2008 survey^9^ reported that nationally 29.5% of learners and 24.4% of black adolescents had ever smoked cigarettes in their lifetime (with 21.0% nationally and 24.5% on at least more than one day in the month prior to the interview). The USYRBS2011 survey^4^ reported that 44.7% of adolescents nationally and 39.1% of black adolescents had ever tried smoking cigarettes (even a puff or two) in their lifetime (with 18.1% nationally and 10.5% on at least more than one day in the month prior to the interview). The current study may be identifying the emergence of alcohol use and cigarette smoking amongst Basotho adolescents, although the problem at this stage does not seem to be as outspoken as in surrounding South Africa or other developed countries. Under-reporting or failure to disclose drinking and smoking habits during a face-to-face interview in the current study, as opposed to anonymous questionnaires used in other surveys^4,8,9^ could, however, have been a limitation.

Taken together, the lifestyle habits in the current study indicate that a transition toward a more sedentary lifestyle, as well as early onset of alcohol use and cigarette smoking, both of which are conducive to weight gain and likely to track into adulthood, may be emerging amongst Basotho adolescents, who are represented in this study by 16-year-old adolescents.

Similarly, analysis of the dietary habits shows a nutritional transition taking place amongst these adolescents. Traditional African diets described in the 1950s were associated with low risk for non-communicable diseases.^38^ Amongst South Africans, the nutritional transition associated with rapid urbanisation, which has been taking place over the last few decades, is typically characterised by increased intake of total fat, animal protein, sugar and salt; and decreased intake of plant protein, dietary fiber and other complex carbohydrates.^38^ The Basotho adolescents in this study reported that maize porridge and bread were still the staple foods which are consumed, almost to the exclusion of most other nutritious foods, by most adolescents on a daily basis (Tables 2, 3 and 4). Fruits and vegetable intakes were very low with only a 10th of the participants consuming at least two servings of fruit and three servings of vegetables per day (Table 2) and only a third reporting that they include vegetables and fruit in their diet on a daily basis (Table 3). Traditional wild vegetables and fruits were consumed only rarely. Vegetables and fruits are sources of vitamins, minerals, fiber and numerous phytochemicals which protect against non-communicable diseases;^39^ adequate intake of fruits and vegetables has also been linked inversely to the risk of overweight and/or obesity in adults and children.^40^

Observational studies demonstrate a significant inverse association between dairy or calcium intake and body weight, body fat or BMI.^41^ Mechanisms for this association may include stimulation of calcium influx into adipocytes, activation of lipogenesis and inhibition of lipolysis by increased calcium; as well as by reduction of fat absorption and increased faecal fat excretion, which are caused by calcium forming insoluble soaps with dietary fat.^42^ Reaching an optimum peak bone density (at around the age of 30) is also considered the best way to counteract the early onset of osteoporosis. Consuming an adequate amount of milk or dairy products is considered the most practical, effective and relatively inexpensive and safe way (when compared with the cost of and risk reported for calcium supplement use) to acquire adequate calcium in order to optimise bone density.^42^ Dairy intake was, however, very low in this population, with more than 90% of participants not consuming the required two to three servings per day. Furthermore, only one in five participants reported consuming dairy on a daily basis whilst 15% reported that they never consume dairy products. The high level of lactose intolerance amongst blacks people in sub-Saharan Africa^43^ may prevent the use of fresh milk; but the intake of fermented milk, which forms part of traditional diets, was also low (Table 4).

The non-quantified FFQ (Table 3) also indicates frequent intake of fatty processed meats, sugary drinks and tuck-shop treats, with low intake of all traditional foods. These trends were not reflected clearly in the 24-hour recall data and in the relatively low total energy intakes calculated from the 24-hour recall data (Table 2). This may reflect what is referred to as the 'flat-slope syndrome’ in which participants with high actual consumption often underestimate their intake of food and drink on recall.^18^

The adolescents showed good basic knowledge about nutrition (average knowledge score of 75%) and overall they portrayed a negative attitude toward overweight and/or obesity, as well as an appreciation for the important role that sound nutrition plays in a healthy lifestyle. Their knowledge regarding the role of overweight/obesity in health was, however, lower (average score of just under 60%). The most concerning finding was the substantial proportion of adolescents who have certain misperceptions regarding nutrition and obesity, as was clear from the responses (Table 5 and 6). These misperceptions highlight areas that need to be targeted through educational programmes in order to address the emerging problems of overweight/obesity amongst adolescents in Lesotho.

### Implications and recommendations

The results of the current study amongst 16-year olds in urban Maseru raise awareness of the fact that Lesotho may be faced with an emerging health issue of adolescent overweight and/or obesity, a problem which is known to track into adulthood and which contributes significantly to morbidity and mortality in later life.^2,3^ With public health and nutrition-related efforts in Lesotho to date having been focused largely on the prevention of undernutrition, these results highlight the need to intervene with educational programmes targeted at school children so as to increase nutritional knowledge and to prevent overweight/obesity. As the current study (for logistical reasons) only included 16-year-old adolescents attending school in Maseru, this survey needs to be repeated on a larger scale to represent all children below 18 years from all parts of Lesotho; and should be designed to compare urban and rural data regarding the prevalence of dietary and lifestyle risk factors of weight gain.

## Conclusion

The current study, which is the only one as yet to perform an investigation specifically looking at overweight and/or obesity amongst adolescents in Lesotho, finds evidence of transition towards westernised dietary patterns characterised by high sugar and fast-food intakes and low fruit, vegetable and dairy consumption, as well as increased sedentary behaviour, amongst 16-year-old school goers in urban Maseru. Such a transition, if occurring amongst Basotho adolescents, puts them at risk for adult obesity and associated non-communicable diseases which will increase the healthcare burden on a low-income developing country with existing high levels of poverty. These data raise awareness of the emerging problem of overweight and/or obesity and the related risk factors amongst the youth in developing countries where emphasis is still placed only on the prevention of undernutrition.
